# Coherent perfect absorption in deeply subwavelength films in the single-photon regime

**DOI:** 10.1038/ncomms8031

**Published:** 2015-05-20

**Authors:** Thomas Roger, Stefano Vezzoli, Eliot Bolduc, Joao Valente, Julius J. F. Heitz, John Jeffers, Cesare Soci, Jonathan Leach, Christophe Couteau, Nikolay I. Zheludev, Daniele Faccio

**Affiliations:** 1 Institute for Photonics and Quantum Sciences and SUPA, Heriot-Watt University, Edinburgh EH14 4AS, UK; 2 Centre for Disruptive Photonic Technologies, Nanyang Technological University, Singapore 639798, Singapore; 3 Optoelectronics Research Centre & Centre for Photonic Metamaterials, University of Southampton, Southampton SO17 1BJ, UK; 4 Department of Physics, University of Strathclyde, Glasgow G1 1XQ, UK; 5 CINTRA CNRS-NTU-Thales, UMI 3288, Singapore, Singapore; 6 Laboratory for Nanotechnology, Instrumentation and Optics, ICD CNRS UMR 6281, University of Technology of Troyes, Troyes, France

## Abstract

The technologies of heating, photovoltaics, water photocatalysis and artificial photosynthesis depend on the absorption of light and novel approaches such as coherent absorption from a standing wave promise total dissipation of energy. Extending the control of absorption down to very low light levels and eventually to the single-photon regime is of great interest and yet remains largely unexplored. Here we demonstrate the coherent absorption of single photons in a deeply subwavelength 50% absorber. We show that while the absorption of photons from a travelling wave is probabilistic, standing wave absorption can be observed deterministically, with nearly unitary probability of coupling a photon into a mode of the material, for example, a localized plasmon when this is a metamaterial excited at the plasmon resonance. These results bring a better understanding of the coherent absorption process, which is of central importance for light harvesting, detection, sensing and photonic data processing applications.

## Abstract (web summary)

Understanding the nature of coherent absorption is essential for exploiting it in new technologies. Here, the authors show that a metamaterial can deterministically absorb photons from a travelling wave with nearly unitary probability, down to the single-photon level.

## Graphical abstract

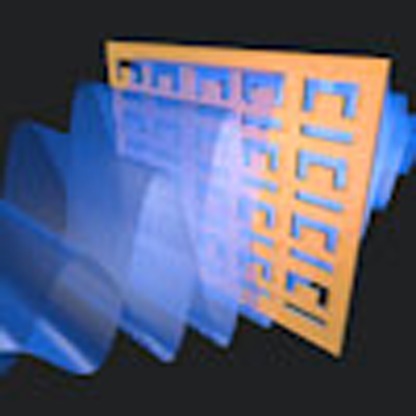

Recent studies provided unexpected but strong evidence that the quantum properties of light are conserved when photons are converted into surface plasmon polaritons, paving the way for active and ultrafast quantum plasmonic technologies^1^,^2^,^3^,^4^,^5^,^6^,^7^ and stimulating a broad interest for the topic. At the same time, light interaction with nanostructured and nanotextured materials exploiting plasmonic resonances is a rapidly growing field of research with potential applications in photovoltaics^8^, water photocatalysis^9^,^10^, artificial photosynthesis^11^,^12^, light harvesting for heating and light sources with engineered emissivity^13^. Furthermore, plasmonics plays a crucial role in the electromagnetic coupling of molecules to quantum dots and metal nanoparticles or nanowires^4^,^14^. Different schemes have been developed to achieve strong coupling of light to metamaterials^15^,^16^,^17^,^18^ including the coherent perfect absorption that was first demonstrated in slabs of lossy materials^19^,^20^,^21^. Light-with-light modulation based on the coherent perfect absorption in metamaterial films of subwavelength thickness is also possible and it has now been demonstrated with a continuous wave laser^22^ and with femtosecond optical pulses exhibiting modulation bandwidths of a few terahertz and possibly beyond^23^. In this process, two coherent beams of light interact on a layer of plasmonic metamaterial in such a way that one beam modulates the intensity of the other. The interference of the two beams can eliminate the plasmonic Joule losses of light energy in the metamaterial with full transmission of the incident light. Depending on the mutual phase of incident beams, it can also lead to the total absorption of light. This provides a method to go beyond the theoretical limit of 50% absorption in a thin film^22^, but also a new way for controlling optical signals^24^. In this work, we explore the mechanisms of coherent absorption at the single-photon level in deeply subwavelength films. We demonstrate that a single photon can be coupled to a plasmon mode of a metamaterial or absorbed in a multilayered graphene film with nearly 100% probability.

## Results

### Coherent control in the single-photon regime

**Figure 1:**
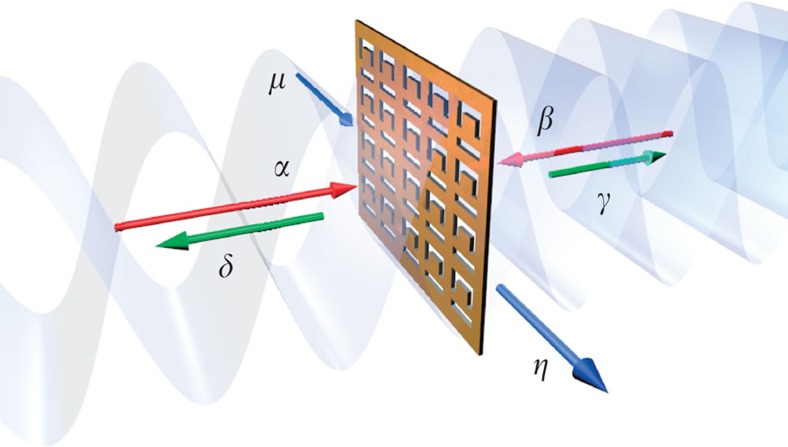
Schematic of metamaterial input and output ports. Interaction of two coherent beams in a thin absorber, here represented as a plasmonic metamaterial: the film is described as a lossy beam splitter with two input photon channels *α* and *β*, two photon output channels *γ* and *δ* and plasmon input and output channels *μ* and *η*.

In a classical wave optics description, a thin absorbing film of subwavelength thickness experiences no interaction with light if it is placed in the node of a standing wave formed by two counter-propagating coherent waves of the same amplitude (for example, obtained using a 50/50 beam splitter). Indeed, the electric field of the light has zero amplitude in the node making no contribution to dipole interactions in the medium. In contrast, the film absorbs strongly in the antinode of the standing wave where the magnitude of the oscillating field is at maximum. A film that absorbs 50% for the travelling wave will absorb 100% in the antinode of the standing wave^22^,^25^. In the quantum regime, the absorber can be treated as a device with two input photon ports, *α* and *β*, and two output photon ports, *γ* and *δ*. We also assume that absorption is related to the excitation of a mode in the material, for example, a plasmonic mode in the case of a metamaterial thus adding two more ports *μ* (input) and *η* (output) to the description of the absorption process, see Fig. 1.

In the regime of linear optics, the output photonic states *γ*, *δ* and plasmonic states *η* are a linear combination of the input states *α*, *β* and *μ*:


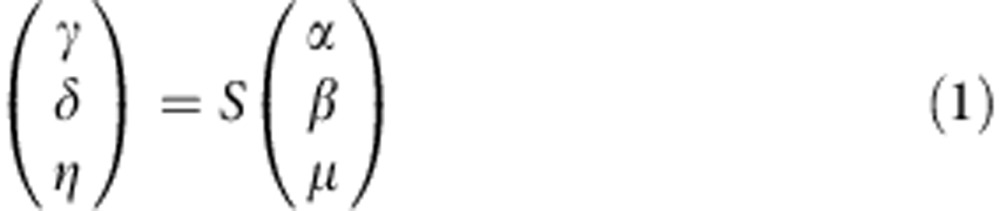


where *S* is the scattering matrix that relates the input to the output states and is given explicitly in the Methods.

### Experiments with single-photon Fock states

**Figure 2:**
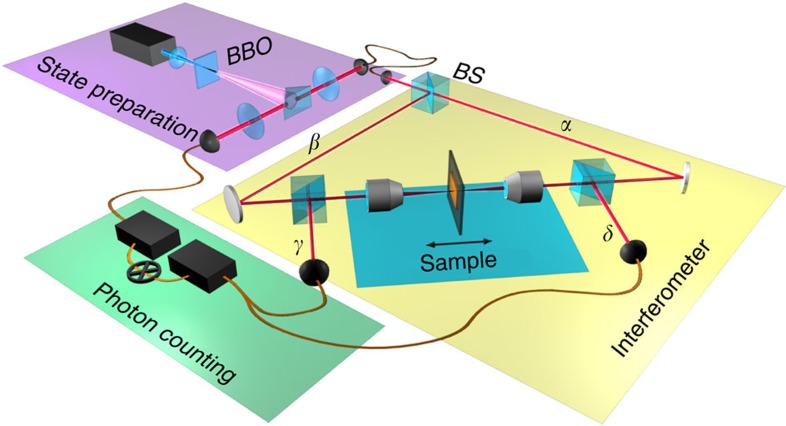
Single-photon experiment on perfect coherent absorption. Illumination of a type 1 beta-barium borate (BBO) crystal by a continuous wave *λ*=405 nm laser producing correlated single photon pairs by SPDC. The correlated photon pair are separated by a knife edge prism. One photon of the correlated pair is used to herald the presence of the other photon that is launched into the interferometer. The metamaterial absorber is placed in the middle point of the interferometer and translated along the optic axis by a piezoelectrically actuated stage. The single photons are focussed onto the sample by × 10 objectives. Photons are then detected in coincidence with the heralding photon at outputs *δ* and *γ*.

In the experiment (layout shown in Fig. 2), a single photon launched into the interferometer via a lossless 50/50 beam splitter generates a coherent superposition state at a metamaterial film. This state may be written as 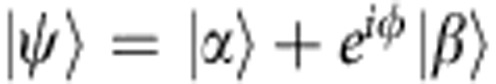, where *φ* is the phase shift between the two input channels. By tuning properly the reflectivity and the transmissivity parameters of the scattering process at the metamaterial sample, this state evolves in such a way that the probability to observe at least one photon in either of the output channels *γ* and *δ* is given by the expression, 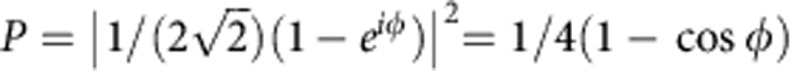. This implies that for *φ*=*π*, no photons will be measured in the output channels and correspondingly the single input photon will be totally absorbed with 100% probability.

**Figure 3:**
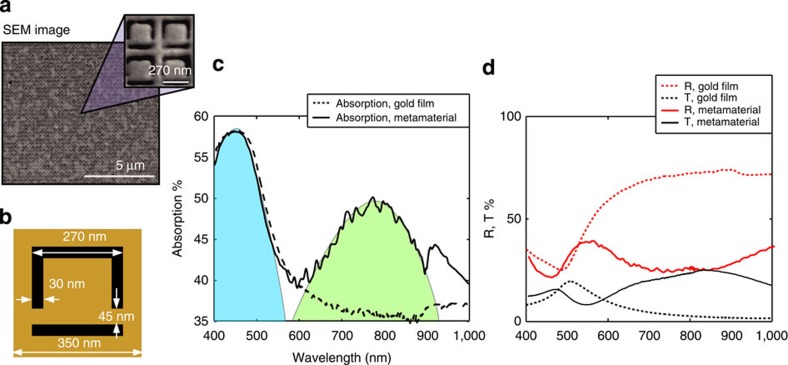
Metamaterial absorber. (**a**) Scanning electron microscope (SEM) image of the milled 50 nm free-standing gold film; (**b**) design of the unit cell; (**c**) the absorption spectrum of the metamaterial film (solid black line). The blue shaded region indicates the natural absorption resonance of gold around 450 nm, while the green shaded region highlights the structure-induced resonance around 810 nm. For comparison, the absorption of an unstructured gold film of the same thickness is also shown (dashed black curve). (**d**) The reflection and transmission curves of the unstructured gold film (dashed curves) and of the metamaterial (solid curves): as can be seen, the structured material exhibits equal 25% transmission and reflection around 800 nm, corresponding also to 50% absorption.

To demonstrate the effect of deterministic single-photon-to-plasmon coupling and thus perfect absorption, we conducted the experiment with a plasmonic metamaterial exhibiting close to 50% travelling wave absorption. The metamaterial absorber was manufactured by focused ion beam milling to form a free-standing 50-nm thick gold film perforated with an array of asymmetric split-ring structures, which provide the desired optical properties congruent with coherent perfect absorption, that is, 50% absorption and equal reflection/transmission amplitudes (see Fig. 3 for details of the sample design and optical characteristics).

The sample was placed at the centre point of the interferometer. Single-photon states were prepared by the spontaneous parametric down conversion (SPDC) of a laser diode with emission line cantered at a wavelength of 405 nm. We used a beta-barium borate crystal producing non-collinear, degenerate entangled photon pairs at *λ*=810 nm. The output ports *γ* and *δ* were monitored with single-photon avalanche detectors (SPADs) gated by the heralding photon channel. The confidence with which a single photon in the interferometer was heralded by a second photon were evaluated in a Hanbury–Brown–Twiss experiment that returned a very high degree of heralded second-order coherence (see Methods), thus indicating a high fidelity of the single photon source. The relative phase shift between the input channels, *φ*, was controlled by translating the metamaterial film along the light propagation direction with a piezoelectric actuator.

**Figure 4:**
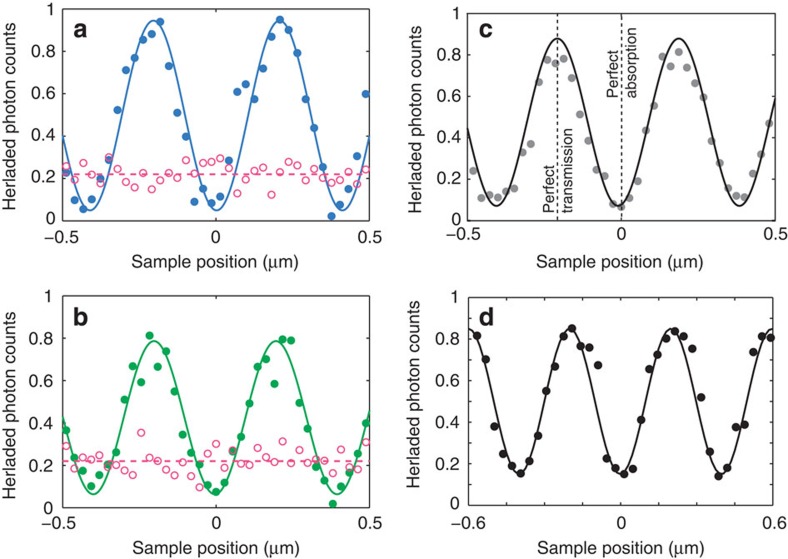
Single-photon perfect coherent absorption. (**a**,**b**) The output photon count rates in channels *γ* and *δ* normalized to the input photon count rates in channels *α* and *β* as functions of the metamaterial absorber position along the standing wave (round, full symbols). Also shown are the results of measurements when the input channel *β* is blocked (open circles and dashed horizontal fitting lines). (**c**) The half-sum of the normalized rates in channels *γ* and *δ*. The vertical dotted lines indicate the positions of nodes and antinodes, corresponding to almost perfect transmission and absorption regimes, respectively. (**d**) is the same as (**c**) but for a different 50% absorber sample, made of a 30-layer chemical vapour deposition-grown graphene film.

Figure 4a,b shows the output photon count rate in channels *γ* and *δ* normalized to the input photon count rate in channels *α* and *β* (measured on the same detectors by removing the sample) as functions of the plasmonic absorber position. With a single photon entering the device at a time, we observe periodic oscillation in the output photon count rate as the phase shift *φ* between two input channels changes. The oscillation period of 405 nm corresponds exactly to the *λ*/2 period. Here perfect absorption corresponds to the minima of the curve. The overall modulation (Fig. 4c) was measured to be between 90 and 10%—the shortfall from a 100% modulation is explainable by a diffusive scattering from the metamaterial film fabrication imperfections and a contribution of quadrupole transitions to the absorption spectra of the split-ring metamaterial^26^. Indeed, the efficiency of the quadrupole absorption is proportional to the gradient of the electric field of the standing wave that reaches maxima at its nodes and thus the presence of quadrupole absorption prevents a total collapse of dipole absorption at these positions.

### 50% absorbing metamaterial

We stress that the optical response of the asymmetric split ring array is in general quite complex. Apart from the dominant dipole contribution of the optical response, there is also a dependence on the magnetic and electric quadrupole modes of the metamaterials resulting in the Fano-type absorption spectrum. This has been studied in a number of papers such as the one given in ref. 26. *De facto*, the electric dipole response generates a different phase shift for transmitted and reflected waves in comparison with the magnetic and quadrupole responses. As a result of interferences of these responses, the pattern of absorption maxima and minima will be shifted with respect to the antinodes and nodes of the standing wave. However, the effect of perfect absorption will still be observed in much the same way as it would be observed for a metamaterial with solely dipolar response.

Finally, our set-up can be converted for the study of absorption from a travelling wave by blocking the input channel *β*. In this case, we register a position-independent level of the normalized photon count rate in the output channel *γ*, which indicates the probability of photon absorption (open circles in Fig. 4a). Indeed, by removing the which-path ambiguity, we are left with only the *α*-state: the output signal *γ* is simply determined by the travelling wave transmission coefficient of the metamaterial. We obtain similar results by monitoring the output port *δ* (open circles in Fig. 4b).

## Discussion

We note that absorption in the free-standing metamaterial film has a predominantly plasmonic nature related to its nanostructuring. Therefore, the coherent absorption process implies a nearly 100% efficient coupling of the single photon to the plasmonic mode of the absorber. Hence, our experiment shows that although the absorption process of a single photon in a travelling wave is by its very nature probabilistic, absorption can be made completely deterministic by providing a which-path ambiguity of the standing wave.

It is important to note that the results shown here can be replicated with any material that exhibits the fundamental properties of 50% absorption and deeply subwavelength thickness. To this end, we repeated the experiment with a 30-layer graphene film, which, as can be seen in Fig. 4d, delivered very similar results.

Our findings also expose the underlying quantum mechanism of optical gating via the coherent absorption process, which has previously been reported with continuous wave and pulsed signals at classical light levels^22^,^23^. The fact that modulation of light can be demonstrated with a single photon proves that the effect of modulation here does not rely on one photon modulating another, for example, via a non-linearity of the film. Rather, the coherent absorption gate exploits a difference in the absorption probabilities between the two configurations of the gate when the control beam is blocked or open. At higher photon fluxes (that is, at classical light levels), this takes the form of an interference-controlled redistribution of the energy flow between the inputs, outputs and the dissipative channel provided by the 50% absorber^27^. In contrast with a gate based on the materials' non-linearity, the coherent absorption gate operates with no harmonic distortion and works at any intensity level.

In conclusion, we have demonstrated experimentally that the coherent absorption process in a thin absorber holds at the single quantum level and that a single photon can be deterministically coupled to a plasmonic mode of a metamaterial. More specifically, our results explicitly show that the same coherent absorption observed in the classical regime can also be observed with single photons. This paves the way for a number of applications ranging for example from the development of single photon sensors in ultrathin film materials to highly efficient coupling of single photons to single plasmons for applications in quantum plasmonics.

## Methods

### Experimental arrangement

Single-photon states are prepared by SPDC; a 100-mW laser diode centred at *λ*=405 nm (Cobolt MLD 405 nm laser diode module) is used to pump a 3-mm thick type-I beta-barium borate crystal producing non-collinear, degenerate entangled photon pairs via SPDC. The photon pairs are then coupled to single-mode fibres by lenses and collimation objectives. A 10-nm bandpass filter centred at 810 nm is used to isolate the SPDC photons from any residual pump photons and ambient light. One of the fibres is connected directly to the photon counting apparatus (SPAD and National Instruments counting card) and is used to herald the presence of another photon within the interferometer. The other fibre output is coupled to the input of a interferometer. The polarization state of the photons is set via a linear Glan-Taylor polarizer (extinction 10,000:1) before passing a lossless (50:50) non-polarizing beam splitter. The beam splitter divides the single photons into arms *α* and *β* of the interferometer, creating the quantum superposition state.

The counterpropagating photons from path *α* and *β* are tightly focussed onto the metamaterial by × 10 Nikon microscope objectives producing a spot size of ∼5 μm. The sample is placed at the centre of the interferometer, within the coherence length (∼200 μm) of the photons and scanned via a piezoelectrically actuated linear translation stage over a few optical cycles. Following the sample, the photons are decoupled from the interferometer cavity via lossless 50:50 beam splitters and coupled to multimode fibres. The photons are then detected in coincidence with the heralded photon via a second SPAD detector. The two detection arms (*γ* and *δ*), which are of different optical path length, are coupled into a single multimode fibre by means of a beam splitter (not shown in Fig. 2) to measure the photon counts on a single SPAD detector. The output ports, *γ* and *δ*, are measured independently by blocking the beam before each of the fibre couplers or simultaneously with both channels open.

### Measurement of heralded single photon *g* ^(2)^(*τ*)

**Figure 5:**
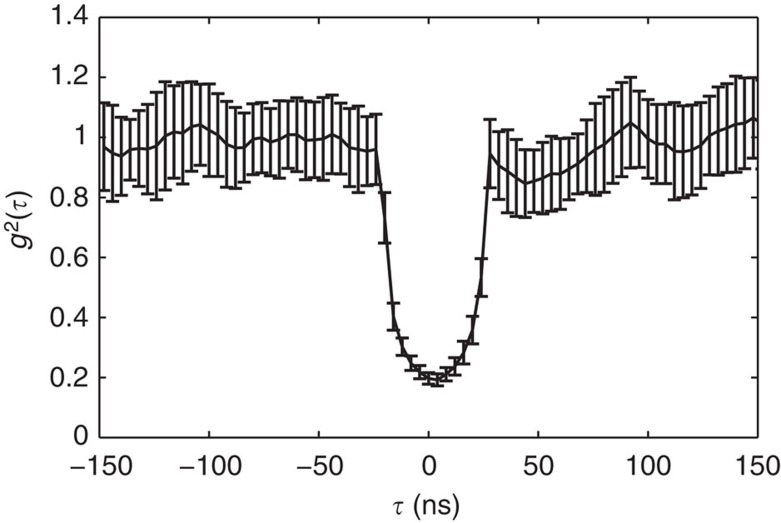
Experimental measurement of the single-photon source *g*^(2)^ function. Measurements are taken with the same 25-ns integration window used in the coherent absorption experiments. The second-order correlation function, *g*^(2)^, is calculated as the mean of the total number of measurements taken at each time delay, *N*=30. Error bars are calculated as the s.d. over the total number of measurements taken.

Figure 5 shows the measured *g*^(2)^(*τ*) for our heralded single-photon source. The standard *g*^(2)^(*τ*) measurement uses three single-photon detectors—a gate detector *D*_*g*_, which is used to herald the presence of a signal, and two detectors *D*_1_ and *D*_2_, one for each output of a beam splitter placed in the path of the signal. A delay of time *τ* can be introduced to the path of detector *D*_2_. The resultant *g*^(2)^(*τ*) is a function of the threefold coincidence rate *R*_12*g*_(*τ*), the twofold coincidence rates *R*_1*g*_ and *R*_2*g*_(*τ*) and the single-event rate *R*_*g*_; thus, it is given by^28^


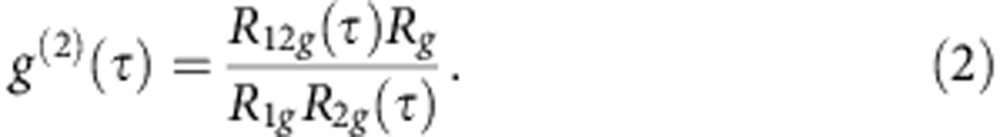


As anticipated for a heralded single-photon source, *g*^(2)^(0)<0.5 and *g*^(2)^(*τ*»0)=1. The width of dip is ∼50 ns, which is due to the 25-ns coincidence detection window and was chosen to be the same as that used in the coherent absorption experiments. The data in Fig. 5 confirm the single-photon nature of our heralded source and thus the quantum nature of the perfect coherent absorption measurements.

### Sample preparation

The free-standing metamaterial structure with 50 × 50 μm overall area was fabricated in a 50-nm gold film by focused ion beam milling. Figure 3 shows a scanning electron microscope image of the sample and its unit cell structure and a comparison between absorption of the unstructured gold film and nanostructured metamaterial film of the same thickness.

### Definition of scattering matrix

The matrix *S* of a infinitely thin absorbing layer has the general form:


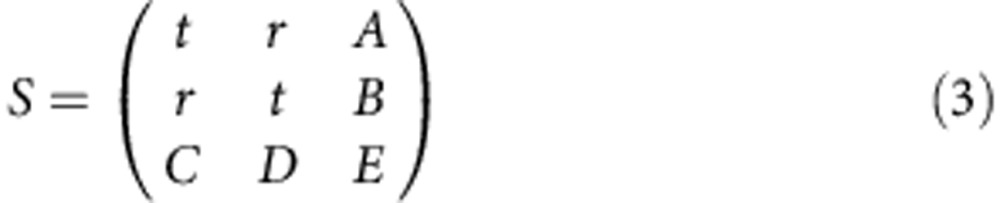


Conservation of energy imposes |*A*|^2^=|*B*|^2^=1–|*t*|^2^–|*r*|^2^. But we also have 2*Re*(tr*)±|*A*|^2^=0, that is, 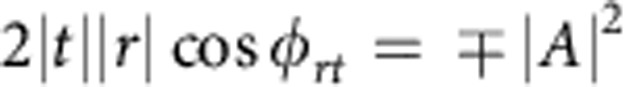, where |*A*|^2^ represents the absorption of the film and *φ*_*rt*_ is the relative phase of *r* and *t*. Combining these equations, we obtain *t*=1±*r* (ref. 18). Combining these relations fixes the maximum absorption for the travelling wave |*A*|^2^=0.5, which is obtained for *t*=±*r* and (keeping only the negative sign term, that is, assuming a *π* phase shift in reflection) *t*=0.5=−*r*. Finally, the S matrix for a thin-film perfect absorber takes the general form:


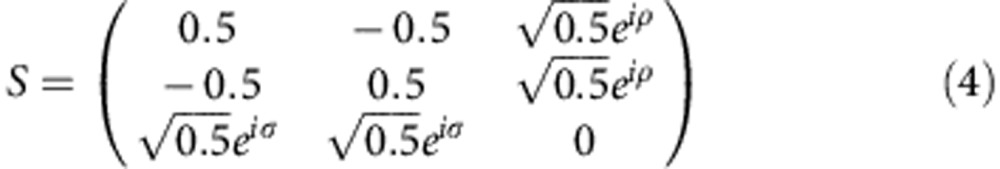


where *ρ* and *σ* are phases related to the excitation/deexcitation of the plasmon mode. Under the assumption that plasmon modes do not decay back into photon modes but are dissipated via non-radiating processes, these phases do not enter the measurable probabilities *P*.

It is also possible to demonstrate that the visibility of the total output energy only depends on the absorption |*A*|^2^. Using the equations above, the single-channel visibility (symmetric thin film) is *V*=[2|*t*||*r*|]/[|*t*|^2^+|*r*|^2^]=[|*A*|^2^]/[(|*A*|^2^–1)cos *φ*_*rt*_]. The total (that is, summing over both output ports, *γ* and *δ*) output visibility is then given by:


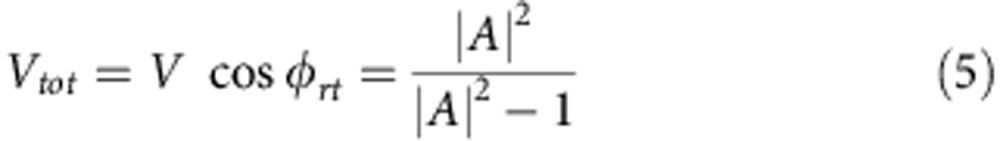


To obtain a visibility >80% as in our case, an absorption >45% is required.

We also explicitly verified numerically (commercial software, COMSOL) that for wavelengths with 50% loss, the metamaterial induces an absolute phase shift on the reflected and transmitted beams of *π* and 0, respectively, in agreement with the conditions *r*=−*t* and |*r*|=1/2 that must be satisfied with deeply subwavelength films and 50% loss^18^.

## Additional information

**How to cite this article:** Roger, T. *et al*. Coherent perfect absorption in deeply subwavelength films in the single-photon regime. *Nat. Commun.* 6:7031 doi: 10.1038/ncomms8031 (2015).
